# Tail Fin Regeneration in Zebrafish: The Role of Non-canonical Crosstalk Between STAT3 and Vitamin D Pathway

**DOI:** 10.7150/ijbs.96400

**Published:** 2025-01-01

**Authors:** Annachiara Tesoriere, Rachele Ghirardo, Francesca Terrin, Francesco Sernesi, Giacomo Meneghetti, Luisa Dalla Valle, Alberto Dinarello, Francesco Argenton

**Affiliations:** 1 Department of Biology, University of Padova, Padua, Italy.; 2 Present address: Novo Nordisk Foundation Center for Stem Cell Medicine (reNEW), University of Copenhagen, Copenhagen DK-2200, Denmark.

**Keywords:** STAT3, Zebrafish, Tail fin regeneration, vitamin D

## Abstract

Stat3 is a transcription factor with a key role in cell proliferation and migration. Using the *stat3^-/-^* zebrafish line we showed that the *stat3* genetic ablation results in a marked decrease of tail fin regrowth, demonstrating that this transcription factor is fundamental in the regeneration process.

Stat3 activity is finely modulated by post-translational modifications that occur in several residues of the protein (i.e., Y705 and S727 phosphorylation), with tissue-specific effects. Using the newly generated *stat3^S→A751^* zebrafish line, we demonstrated that the Stat3 phosphorylation in the non-canonical S751 site (homologous of mammalian serine 727) is required for the regeneration of tail fin in both larval and adult stage, even if this phosphorylation has largely been reported to have marginal roles in Stat3 activity.

Our analysis showed that both *stat3^-/-^* and *stat3^S→A751^* mutant zebrafish lines have alterations in the expression of genes involved in epithelial and bone tissue regeneration, including genes coding for the vitamin D signaling pathway. Interestingly, the reduced regeneration activity in zebrafish *stat3^-/-^* and *stat3^A751/A751^* larvae is partially rescued by vitamin D treatment. Together, these results reveal a Stat3-vitamin D co-regulatory mechanism during zebrafish tail fin regeneration.

## Introduction

The signal transducer and activator of transcription (STAT) 3 is a transcription factor that controls the expression of target genes modulating immune response, stem cell self-renewal, metabolism and cell proliferation 1-3. Historically, STAT3 activity has been related to interleukin (IL) 6 family member proteins, which, binding their membrane receptors, trigger the phosphorylation of Janus kinases (JAK) 1/2/3 and, consequently, the phosphorylation of STAT3 at the level of tyrosine 705 (pY705)^4^. Although pY705 is still considered the main mechanism of STAT3 activation, other post-translational modifications are currently gaining additional attention in the study of STAT3 biology 5. For instance, Serine 727 phosphorylation (pS727) has gradually become more relevant in the analysis of the canonical and non-canonical functions of STAT3. pS727 can indeed regulate the transcription of several target genes inducing or repressing pY705-dependent genes according to the cellular context 5-7. Additionally, pS727 is fundamental for the induction of the mitochondrial activities of STAT3 8-11. Whereas murine *Stat3* knockout (KO) is lethal 12, the S727-specific mutant mouse (named *Stat3^SA/SA^*) did not show overt defects when compared to wild type (WT) siblings 13. Nevertheless, *Stat3^SA/SA^*-derived fibroblasts showed a reduced responsiveness to IL-6 when compared to WT, corroborating the role of S727 in the proper functions of STAT3 13.

In zebrafish, Stat3 is well conserved and shows high similarity with human and murine STAT3. Moreover, the zebrafish *stat3* KO leads to several developmental and transcriptional defects and is not compatible with adult life 10,14,15. Noteworthy, the few *stat3* KOs that can reach later stages of development (2-3 months post fertilization, mpf) show severe defects in body axis elongation and spinal cord formation 14-17. Notably, *stat3* KO phenotype recapitulates some of the symptoms observed in humans affected by autosomal dominant hyper IgE syndrome (AD-HIES), a heterogeneous group of immunodeficiency disorders mainly caused by STAT3 deficiency 16,18,19. AD-HIES is related to severe defects in tissue damage regeneration and wound healing 20, and zebrafish represents a suitable platform to study these latter processes 21. Therefore, we decided to use zebrafish as a model to study AD-HIES and to test tail fin regeneration and the regeneration-dependent gene expression in *stat3^ia23/ia23^* KO zebrafish model (hereafter named *stat3^-/-^*)^14^ and in newly generated *stat3^A751/A751^* zebrafish, which bears a mutation that converts S751 (homologous of murine and human S727) with an alanine.

Moreover, our aim was to pinpoint a possible drug that could rescue these abrupt phenotypes, and we identified vitamin D as a putative candidate.

Vitamin D exerts regenerative activities in zebrafish cardiac tissue 22, bone tissue 23 and fin 24. The most relevant biochemical processes that lead to vitamin D maturation and biological activities are conserved among vertebrates 25. The conversion of pre-vitamin D into mature vitamin D and its inactivation rely on the activity of several mitochondrial enzymes (CYP2R1, CYP27B1 and CYP24A1). The mature vitamin D interacts with its specific receptor, called vitamin D receptor (VDR) that exerts genomic (acting as a transcription factor) and non-genomic functions (binding on the proteins of plasma membrane and activating phosphatases, kinases and ion channels) 26-29.

The main enzymes involved in vitamin D metabolism in human are well conserved in zebrafish: the zebrafish genes* cyp27b1* and *cyp24a1* encode for the enzymes that catalyze respectively the synthesis and degradation of active vitamin D, while the two paralog genes *vdra* and *vdrb* recapitulate the functions of human VDR. Taking advantage of this similarity, we decided to study vitamin D involvement in the Stat3-mediated regeneration.

Our results demonstrated that the Stat3 S751 is necessary for tail fin regeneration in zebrafish. Moreover, we showed that pS727 Stat3 can regulate the vitamin D metabolism, and that this interplay is involved in the regeneration process. In turn, exogenous vitamin D can escape this mechanism, partially rescuing the Stat3-mediated regeneration defects we observed in *stat3^-/-^* and *stat3^A751/A751^* larvae.

## Results

### STAT3 phosphorylations regulate regeneration *in vitro* and *in vivo*

**Figure 1:**
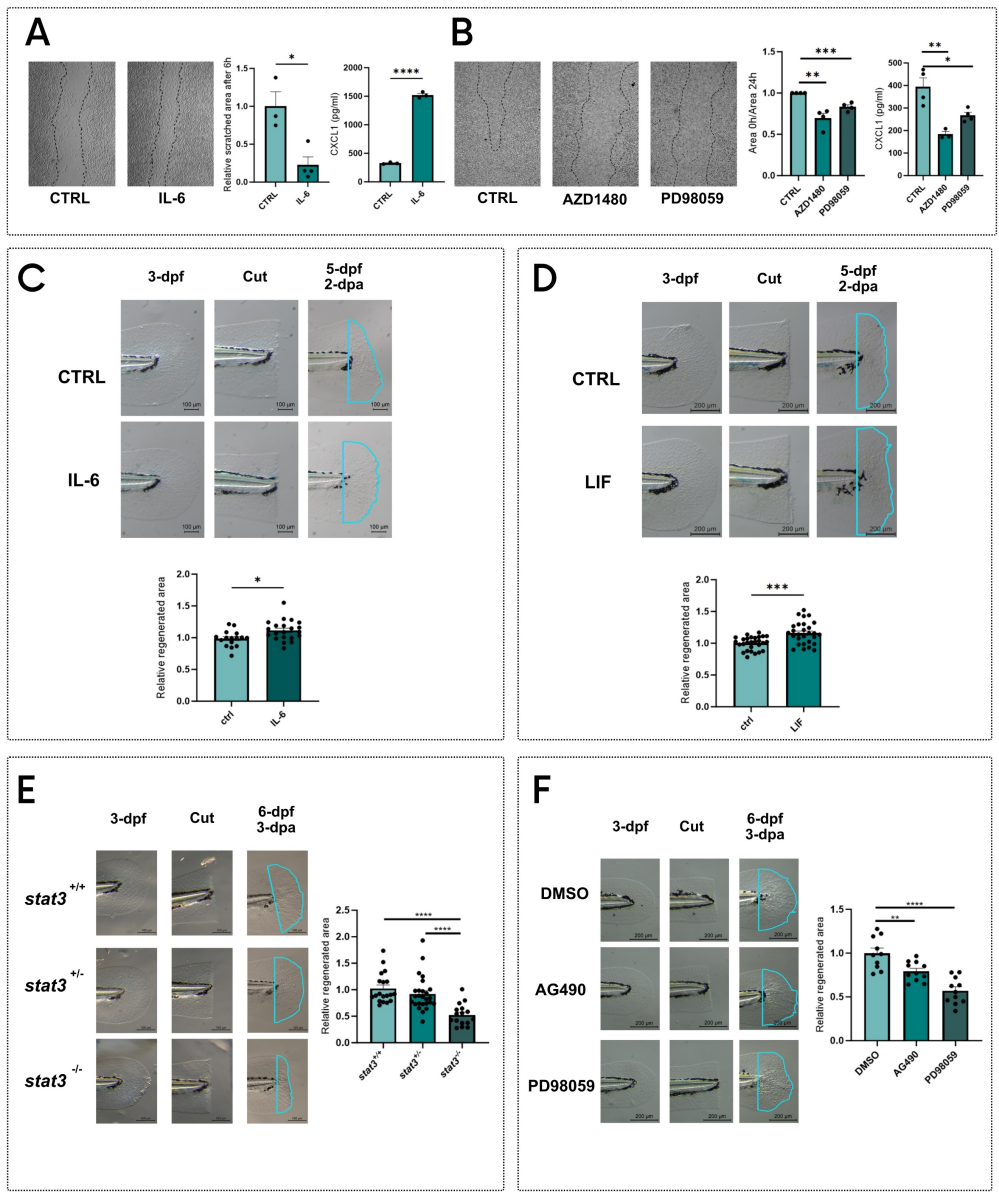
** STAT3 phosphorylations regulate regeneration *in vitro* and *in vivo*. A** Representative pictures, measurements of scratched area and levels of CXCL1 in supernatants of L929 cells after 6 hours of treatment with vehicle and 1 ng/ml IL-6. **B** Representative pictures, measurements of scratched area and levels of CXCL1 in supernatants of L929 cells after 24 hours of treatment with vehicle, 5 μM AZD1480 or 12.5 μM PD98059. **C** Representative pictures and quantification of regenerated area of wild type zebrafish tail fins cut at 3-dpf and treated for 2 days either with vehicle or 0.5 ng/ml IL-6. **D** Representative pictures and quantification of regenerated area of wild type zebrafish tail fins cut at 3-dpf and treated for 2 days either with vehicle or 200 nM LIF.**E** Representative pictures and quantification of regenerated area of *stat3^+/+^*^,^ *stat3^+/-^* and *stat3^-/-^* tail fins cut at 3-dpf and analysed at 3 dpa. **F** Representative pictures and quantification of regenerated area of wild type zebrafish fins cut at 3-dpf and treated for 3 days either with DMSO, 50 μM AG490 or 12.5 μM PD98059. Mean ± SEM. *p < 0.05, **p < 0.01, ***p < 0.001, ****p < 0.0001.

The roles of Stat3 in the regulation of cell proliferation and stem cell maintenance 1,7,8 suggest that this transcription factor may be involved in regeneration^30^. To assess *in vitro* the role of STAT3 in regeneration, we performed the scratch test on the murine fibroblast cell line L929. Cells were then stimulated with IL-6 to activate STAT3. After 6 hours of treatment, we observed an almost complete reoccupation of the scratch, which is still visible in control plates (**Figure 1A**). During the regeneration of injuries, fibroblasts communicate with immune cells by secreting chemokines 31, among which CXCL1 plays a central role in wound healing 31-33. For this reason, we measured the CXCL1 levels in the supernatants of L929 cells treated with vehicle or IL-6 (**Figure 1A**), observing an upregulation of CXCL1 production after the treatment. Additionally, to disentangle whether this effect relies on pY705 or pS727, we treated L929 cells with either AZD1480 32,33 or PD98059 9,10,14, which are specific inhibitors of STAT3 phosphorylation and prevent the pY705 and pS727, respectively. Both treatments hamper scratch regeneration compared to untreated scratched cells (**Figure 1B**). To confirm these results with other cell lines, we used the murine B16F10 and the human 1205Lu melanoma cell lines: in both cases AZD1408 significantly blocks scratch reoccupation, whereas PD98059 significantly inhibits cell proliferation in the injured area only in B16F10 cells (**Figure S1**). Notably, we saw a significant downregulation of CXCL1 production in L929 cells treated with AZD1408 and PD98059 compared to controls. (**Figure 1B**).

To corroborate our results, we decided to test STAT3 phosphorylation effects on regeneration *in vivo*. Since the *Stat3* gene is highly conserved among vertebrates, with 88% of homology between *Homo sapiens* and *Danio rerio* (**Figure S2**), we decided to use zebrafish as a model organism to study STAT3 roles in tissue regeneration. Zebrafish tail fin can indeed completely regenerate within 3 days in larvae and 15-20 days in adults and represent a valuable model to study the molecular mechanisms determining tissue regeneration 34-37. We cut tail fins of 3-day post fertilization (dpf) zebrafish larvae and we incubated them with 0.5 ng/ml mouse IL-6 for 48 hours. Notably, two days after amputation (dpa), IL-6-treated larvae showed a significant increase in regenerated tail fin area when compared to control larvae, confirming the results obtained with IL-6-stimulated fibroblasts (**Figure 1C**). We also tested another JAK/STAT3 agonist, the Leukemia inhibitory factor (LIF). Similarly to what we observed with IL-6, we saw a significant increase of tail fin regeneration upon LIF treatment for 48 hours (**Figure 1D**).

These effects were further complemented using the *stat3* knockout zebrafish line formerly generated in our laboratory 14. Interestingly, *stat3^-/-^* 6-dpf larvae showed a marked reduction in tail fin regeneration when compared to WT and heterozygous siblings (**Figure 1E**).

In parallel, we decided to chemically inhibit pY708 (homologous of murine and human Y705) or pS751 in zebrafish larvae (homologous of murine and human S727). We took advantage of the Stat3 specific inhibitors AG490 and PD98059, which prevent the pY705 and pS727, respectively and have been already tested on zebrafish 14,16,38. We treated 3-dpf WT zebrafish larvae with these inhibitors after tail fin clipping. Interestingly, both AG490 and PD98059 significantly reduced the regenerated area of the tail fin after 3-dpa (**Figure 1F**), suggesting that both pY705 and pS727 play a key role in tissue regeneration. To further confirm our results, we also tested the effects of AZD1480 as an alternative pY705 inhibitor on tail fin regeneration. Results shown in **Figure S3** demonstrate a significant downregulation of regeneration upon AZD1480 treatment, confirming the phenotype induced by AG490. While the role of pY705 in the regulation of Stat3 activity has already been largely studied, pS727 functions are still under debate. However, PD98059 is a MEK/ERK1-2 inhibitor whose action can affect many off-target molecules and pathways, thus its activity cannot be directly related to S727 phosphorylation. For this reason, we generated a zebrafish mutant line to specifically analyze the role of S727 in the regeneration process.

### Generation and characterization of the zebrafish *stat3 ^S→A751^* line

**Figure 2:**
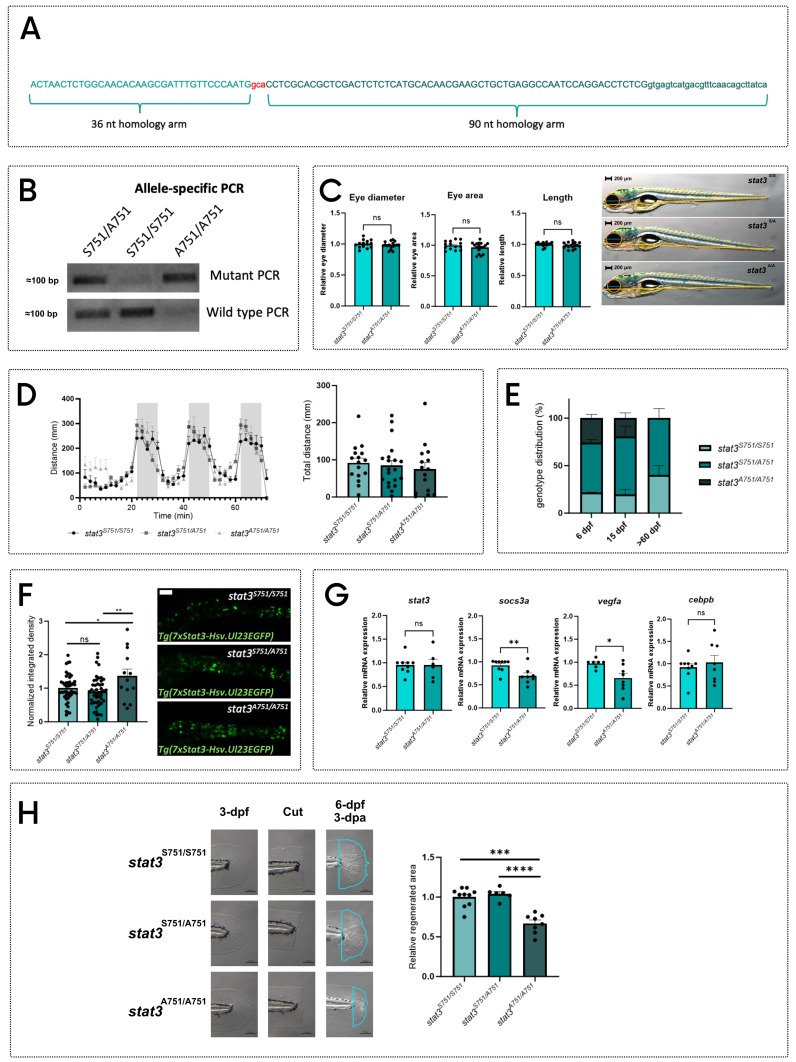
** Generation of *stat3^S→A751^* zebrafish knock-in line. A** Sequence of the donor DNA used to generate the KI. The mutation (gca) is highlighted in red and is flanked by a 36-nt and a 90-nt homology arms. **B** Representative picture of allele-specific PCRs routinely performed to genotype KI fish: heterozygotes are positive for both alleles, whereas homozygotes are positive for only one. **C** Morphological analysis (in blue: eye diameter; in orange: eye area; in yellow: body length) and representative pictures of 6-dpf *stat3^S751/S751^*, *stat3^S751/A751^* and *stat3^A751/A751^* larvae. Scale bar: 200 μm. **D** Standard light/dark behavioral assay performed on 6-dpf *stat3^S751/S751^*, *stat3^S751/A751^* and *stat3^A751/A751^* larvae. **E** Genotype distribution of *stat3^S751/S751^*, *stat3^S751/A751^* and *stat3^A751/A751^* zebrafish at 6 dpf, 15 dpf, and 60 dpf. **F** Fluorescent image of *Tg(7xStat3-Hsv.Ul23:EGFP)^ia28^* intestines of 6-dpf *stat3^S751/S751^*, *stat3^S751/A751^* and *stat3^A751/A751^* larvae and relative fluorescence quantification. Scale bar: 100 μm. **G** Expression levels of *stat3*, *socs3a*, *vegfa* and *cebpb* in 6-dpf *stat3^S751/S751^* and *stat3^A751/A751^* larvae. **H** Regeneration rate of 6-dpf *stat3^S751/S751^*, *stat3^S751/A751^* and *stat3^A751/A751^* larvae at 3 dpa. Mean ± SEM. *p < 0.05, **p < 0.01, ***p < 0.001, ****p < 0.0001.

To study S727 functions we generated a new zebrafish line in which we induced a mutation meant to substitute a TCG codon with a GCA (**Figure 2A**) in the exon 23 of the s*tat3* gene, thus substituting the S751 residue (homologous of mammalian S727, **Figure S2**) with an alanine.

This *stat3^ S→A751^* mutant line can be routinely genotyped with allele-specific PCR (**Figure 2B**). The S751-to-A751 conversion does not affect the first stages of development: no significant morphological alterations were detected in *stat3^A751/A751^* larvae (**Figure 2C**). Moreover, at 6-dpf the mutation does not affect larval behaviour: light/dark transition test did not reveal significant differences in the response to light nor in the total distance swum (**Figure 2D**). However, while mendelian ratios of the three genotypes are maintained at 6- and 15-dpf, we rarely managed to obtain *stat3^A751/A751^* animals from 60-dpf onwards, a result that suggests a strong and unexpected importance of S751 phosphorylation for zebrafish survival (**Figure 2E**).

As a control, we generated another *stat3* zebrafish knock-in line, converting the Serine residue in position 729 of exon 22 (that belongs to a zebrafish-specific region in the transactivation domain, **Figure S2**) to an Alanine (**Figure S4A**). These mutants can also be genotyped by allele-specific PCRs (**Figure S4B**). Homozygous mutants can reach adulthood, are fertile and do not show alterations of their survival rate demonstrating that, while S751 is essential for life, S729 is not.

Since pS727 has tissue-specific functions in the regulation of STAT3 activity, 5,10,15, we wanted to test *in vivo* whether the S751A substitution affects Stat3 functions. To this aim, we analyzed the *stat3^A751/A751^* larvae in transgenic background *Tg(7xStat3-Hsv.Ul23:EGFP)^ia28^* 14. While we could not see any statistical difference in intestinal fluorescence of 6-dpf *stat3^A729/A729^* larvae (**Figure S4C**), *stat3^A751/A751^* 6-dpf larvae displayed a significant upregulation of intestinal fluorescence compared to *stat3^S751/S751^,* suggesting that the abrogation of pS751 determines an upregulation of Stat3 activity in intestinal stem cells (**Figure 2F**). This result was in line with the upregulation we observed in *Tg(7xStat3-Hsv.Ul23:EGFP)^ia28^* larvae treated with PD98059 (**Figure S4D**). To further investigate the role of S751 in the transcription of Stat3 targets, we tested the expression of Stat3 canonical target genes in both *stat3^A729/A729^* and *stat3^A751/A751^* larvae: while the expression of some of these targets was affected in s*tat3^A751/A751^* (**Figure 2G**) no differences were observed in *stat3^A729/A729^* background (**Figure S4E**).

To test our hypothesis on the role of mammalian STAT3 pS727 in the regulation of regeneration, we clipped tail fin of 3-dpf *stat3^S751/S751^*, *stat3^S751/A751^* and *stat3^A751/A751^* zebrafish larvae and evaluated the regeneration rate at 3-dpa. As already demonstrated in *stat3^-/-^* larvae, also *stat3^A751/A751^* larvae showed a significant reduction in the regenerated area, confirming that S751 of Stat3 is required for the correct regeneration of larval fin (**Figure 2H**). Nevertheless, *stat3^A729/A729^* zebrafish larvae do not show evidence of regeneration impairment (**Figure S4F**), confirming the specific role of S751 in the regulation of this process.

### S751 Stat3 regulates the transcription of regeneration-related genes, involved in inflammation, tail fin formation and vitamin D metabolism

**Figure 3:**
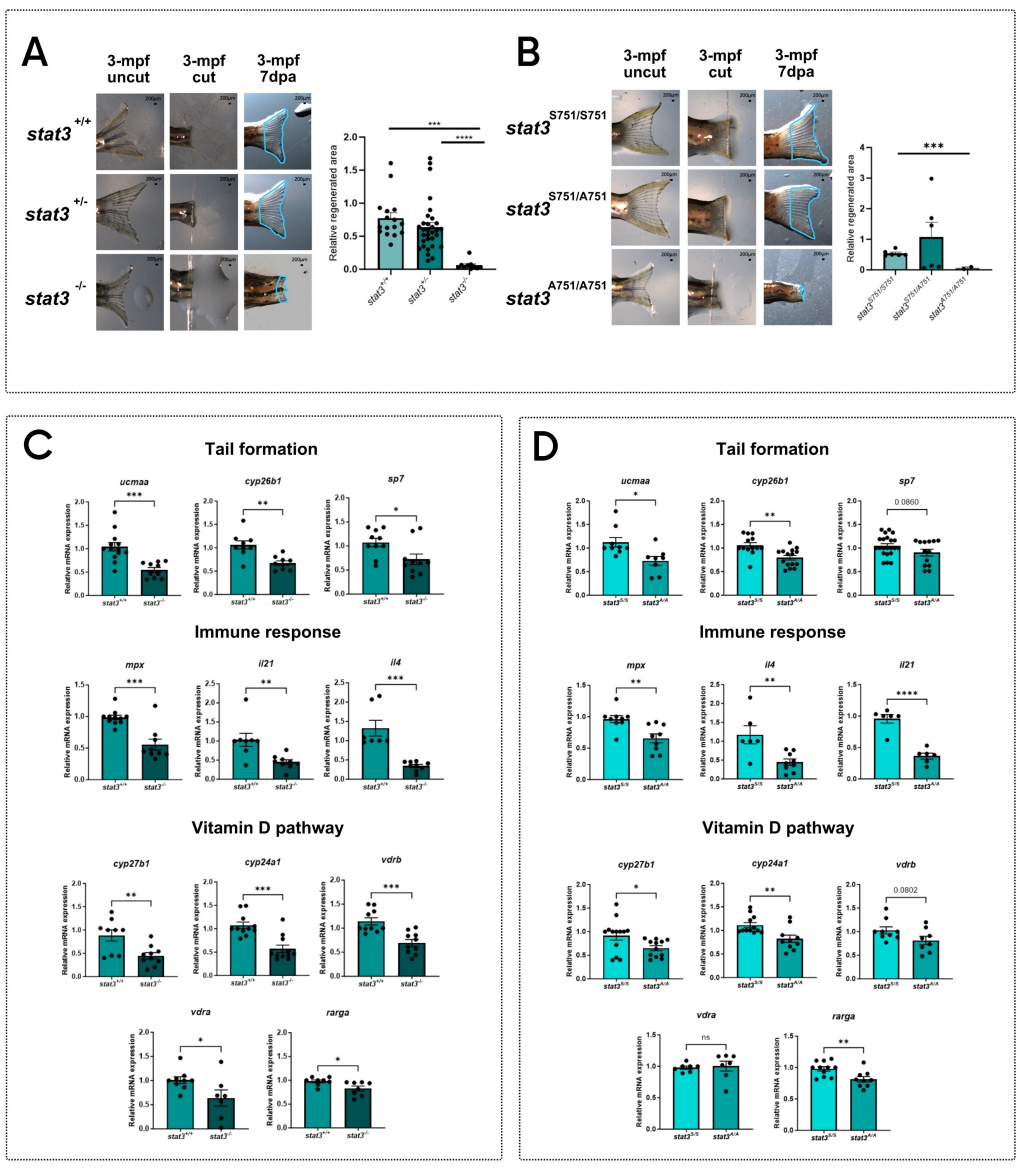
** pS727 Stat3 regulates inflammation, bone metabolism and vitamin D pathway.** Representative pictures of regenerated tail fin of *stat3^+/+^*, *stat3^+/-^* and *stat3^-/-^* adult zebrafish and relative quantification. The adult homozygous mutants are rare escapers that manage to reach this developmental stage. **B** Representative pictures of regenerated tail fin of *stat3^S751/S751^*, *stat3^S751/A751^* and *stat3^A751/A751^* adult zebrafish and relative quantification. The adult homozygous mutants are rare escapers that manage to reach this developmental stage. **C** Expression level of *ucmaa*, *cyp26b1*, *sp7*, *mpx*, *il21*, *il4*, *cyp27b1*, *cyp24a1*, *vdrb*, *vdra*, and *rarga* in *stat3^+/+^* and *stat3^-/-^* larvae. **D** Expression level of *ucmaa*, *cyp26b1*, *sp7*, *mpx*, *il21*, *il4*, *cyp27b1*, *cyp24a1*, *vdrb*, *vdra*, and *rarga* in 6-dpf *stat3^S751/S751^* and *stat3^A751/A751^* larvae. Mean ± SEM. *p < 0.05, **p < 0.01, ***p < 0.001, ****p < 0.0001.

After demonstrating the marked reduction of the regenerative potential in both *stat3^-/-^* and *stat3^A751/A751^* larvae, we analyzed fin regeneration in the adult zebrafish, using the few homozygous mutant escapers that managed to reach the adult stage. Results show that the strong phenotype observed in larvae worsens during aging, with the almost complete absence of regeneration in mutants compared to WT and heterozygous siblings (**Figure 3A-B**).

To better understand the role of Stat3 in this process, we decided to test the expression of a number of regeneration-related genes that are known to regulate tail fin formation (*cyp26b1, ucmaa, sp7*) and the recruitment of immune cells towards the regenerating tissue (*mpx, il4, il21*). All these transcripts are significantly downregulated in both mutants when compared to WT siblings, demonstrating that Stat3 is involved in the expression of these genes and that S751 is also required (**Figure 3C-D**).

Considering that vitamin D is deeply involved in the regulation of immune-response 39,40 and tail formation 41,42, we hypothesized that *stat3* mutations may impact on the vitamin D pathway. Thus, we decided to analyze the level of expression of genes related to the vitamin D metabolism and pathway (*cyp27b1, cyp24a1, vdra, vdrb, rarga*) in *stat3* mutants.

We analyzed the expression of *cyp27b1* and *cyp24a1,* respectively involved in vitamin D synthesis and degradation, showing that they are significantly downregulated in both in *stat3^-/-^* and *stat3^A751/A751^* zebrafish larvae compared to WT siblings (**Figure 3C-D**). We also analyzed the expression level of* vdra* and *vdrb,* observing a significant downregulation in the *stat3^-/-^* larvae but not in the *stat3^A751/A751^* mutants compared to WT siblings.

### Vitamin D improves the regeneration of zebrafish larval tail

Vitamin D is known to improve regeneration, but this effect has been mainly studied in adult zebrafish, so it lacks characterization at larval stages 23.

**Figure 4:**
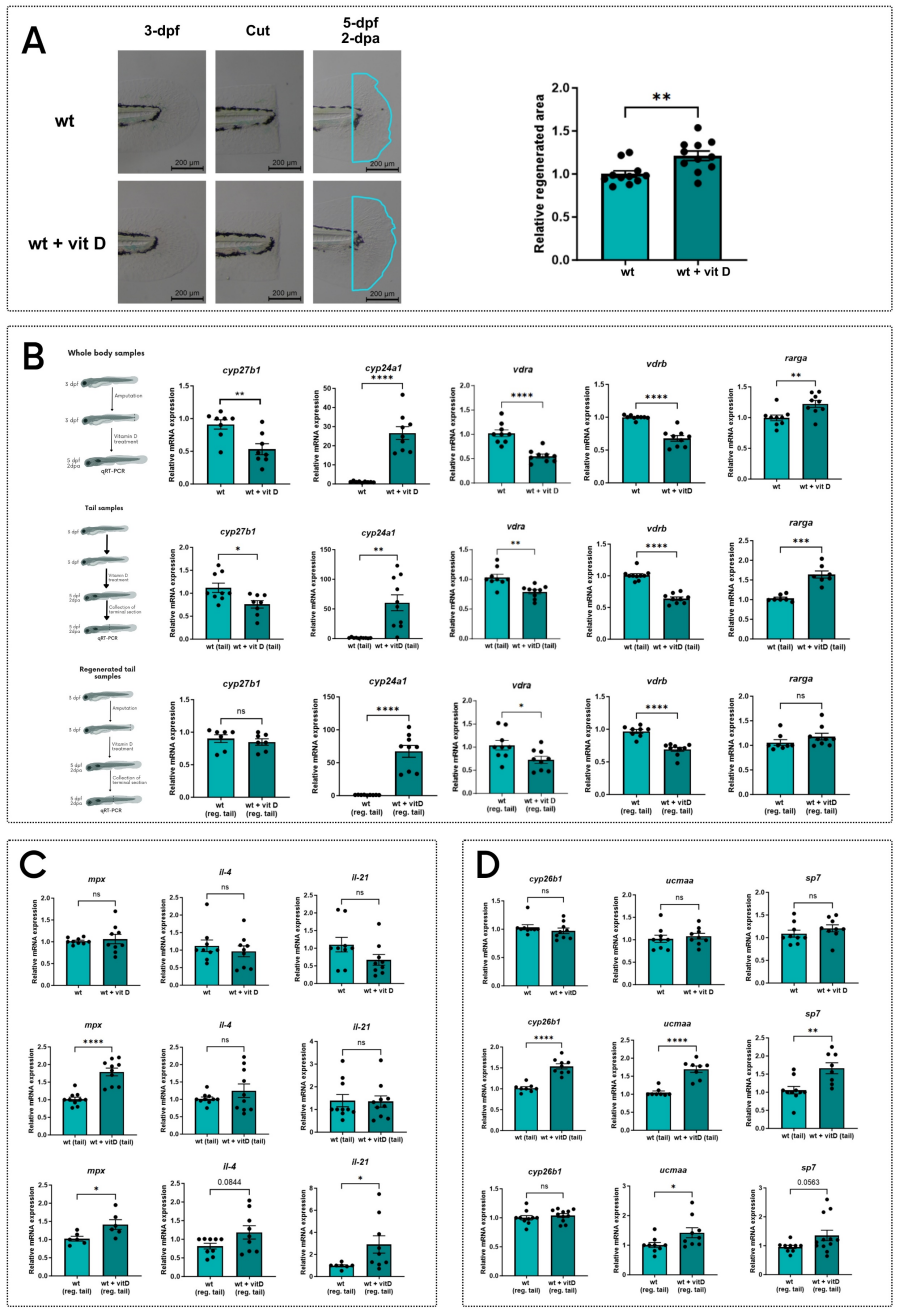
** Vitamin D treatment improves tail fin regeneration. A** Representative pictures and quantification of regenerated area of wild type zebrafish tail fins cut at 3-dpf and treated for 2 days either with vehicle or 0.5 nM vitamin D. **B** Expression levels of *cyp27b1*, *cyp24a1*, *vdrb*, *vdra* and *rarga* in whole body, tail and regenerated tail samples from 5-dpf larvae treated for 2 days either with vehicle or 0.5 nM vitamin D. **C** Expression levels of *mpx*, *il4*, and *il21* in whole body, tail and regenerated tail samples from 5-dpf larvae treated for 2 days either with vehicle or 0.5 nM vitamin D. **D** Expression levels of *cyp26b1*, *ucmaa*, and *sp7* in whole body, tail and regenerated tail samples from 5-dpf larvae treated for 2 days either with vehicle or 0.5 nM vitamin D. Mean ± SEM. *p < 0.05, **p < 0.01, ***p < 0.001, ****p < 0.0001.

To study vitamin D effects on larval tail regeneration, we treated WT 3-dpf larvae with vitamin D upon tail fin clipping and measured the regenerated area after 2-dpa. Vitamin D-treated larvae showed a larger regenerated area compared to the untreated controls (**Figure 4A**).

To evaluate how vitamin D improves larval tail fin regeneration, we analyzed three different sets of RNA samples: whole body samples of 5-dpf larvae treated with vitamin D for 48h upon tail fin clipping at 3-dpf; tail samples of 5-dpf larvae treated with vitamin D for 48h without cutting the tail; regenerated tail samples of larvae treated with vitamin D for 48h after amputation (extensively described in **Figure S5A**). On these samples, we measured the transcriptional levels of the genes previously reported in **Figure 3**, which were downregulated in the *stat3* mutant zebrafish and are reported in literature to be regulated by vitamin D.

Vitamin D upregulates *cyp24a1* gene expression, while *vdra* and *vdrb* are downregulated, probably due to the increased availability of their ligands as negative feedback. These direct effects of the vitamin D treatment are detected in all the three types of samples, confirming the global impacts of vitamin D (**Figure 4B**).

The influence of vitamin D treatment on the genes correlated to the immune system is not evident on the whole body of larvae (**Figure 4C**)*.* However, both uncut and regenerated tail samples showed a significant upregulation of *mpx* when compared to untreated controls*,* demonstrating a tail-specific effect of vitamin D on *mpx* expression. Notably, vitamin D increases *il4* (p=0.0844) and *il21* expression only in regenerated samples (**Figure 4C**), suggesting a vitamin D-mediated effect relative to regeneration.

Additionally, vitamin D treatment significantly upregulates the expression of *cyp26b1*, *ucmaa* and *sp7* in uncut samples, but not in the whole-body samples, suggesting a possible tail-specific activation of these vitamin D-responsive genes. Moreover, the expression of *ucmaa* and *sp7* increases in the regenerated tail samples of larvae treated with vitamin D, compared to controls. These results support our hypothesis that vitamin D promotes the expression of genes involved in cartilage and bone formation during the development and the regeneration of the tail fin (**Figure 4D**).

**Figure 5:**
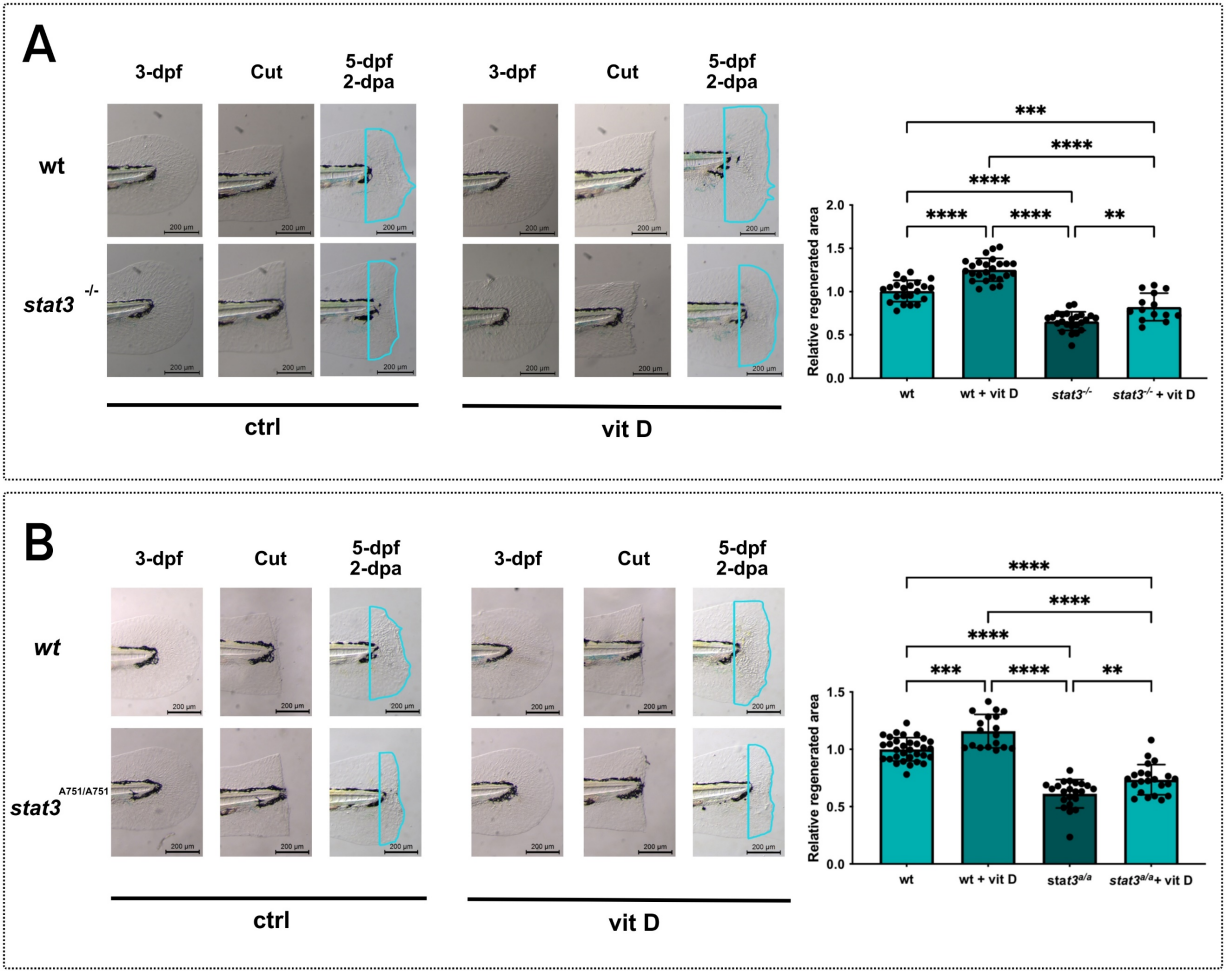
** Vitamin D partially recovers the regeneration impairment of *stat3* mutants. A** Representative pictures and relative quantification of *stat3^+/+^* and *stat3^-/-^* larval tail fins cut at 3 dpf and treated for 2 days with vehicle or 0.5 nM vitamin D. **B** Representative pictures and relative quantification of *stat3^S751/S751^* and *stat3^A751/A751^* larval tail fins cut at 3 dpf and treated for 2 days with vehicle or 0.5 nM vitamin D. Mean ± SEM. **p < 0.01, ***p < 0.001, ****p < 0.0001.

We hypothesized that vitamin D, acting on the same targets of Stat3, could rescue the lack of regeneration in *stat3* mutant lines. Thus, we repeated the vitamin D treatment on 3-dpf *stat3* mutant larvae after tail fin clipping, and we analyzed the regenerated area at 2-dpa. Regeneration in mutant larvae is partially rescued by the vitamin D treatment (**Figure 5A-B**), suggesting that vitamin D to some extent bypasses Stat3-dependent functions in tail fin regeneration.

Altogether, the match between the downregulation of regeneration-related genes in mutant backgrounds and their stimulation by vitamin D treatment and, at the same time, the improvement of regeneration in both *stat3* mutants corroborate the hypothesis of a pS751 Stat3-dependent control on vitamin D pathway.

### 5. Stat3 regulates vitamin D pathway modulating the expression of vitamin D metabolism-related genes

**Figure 6:**
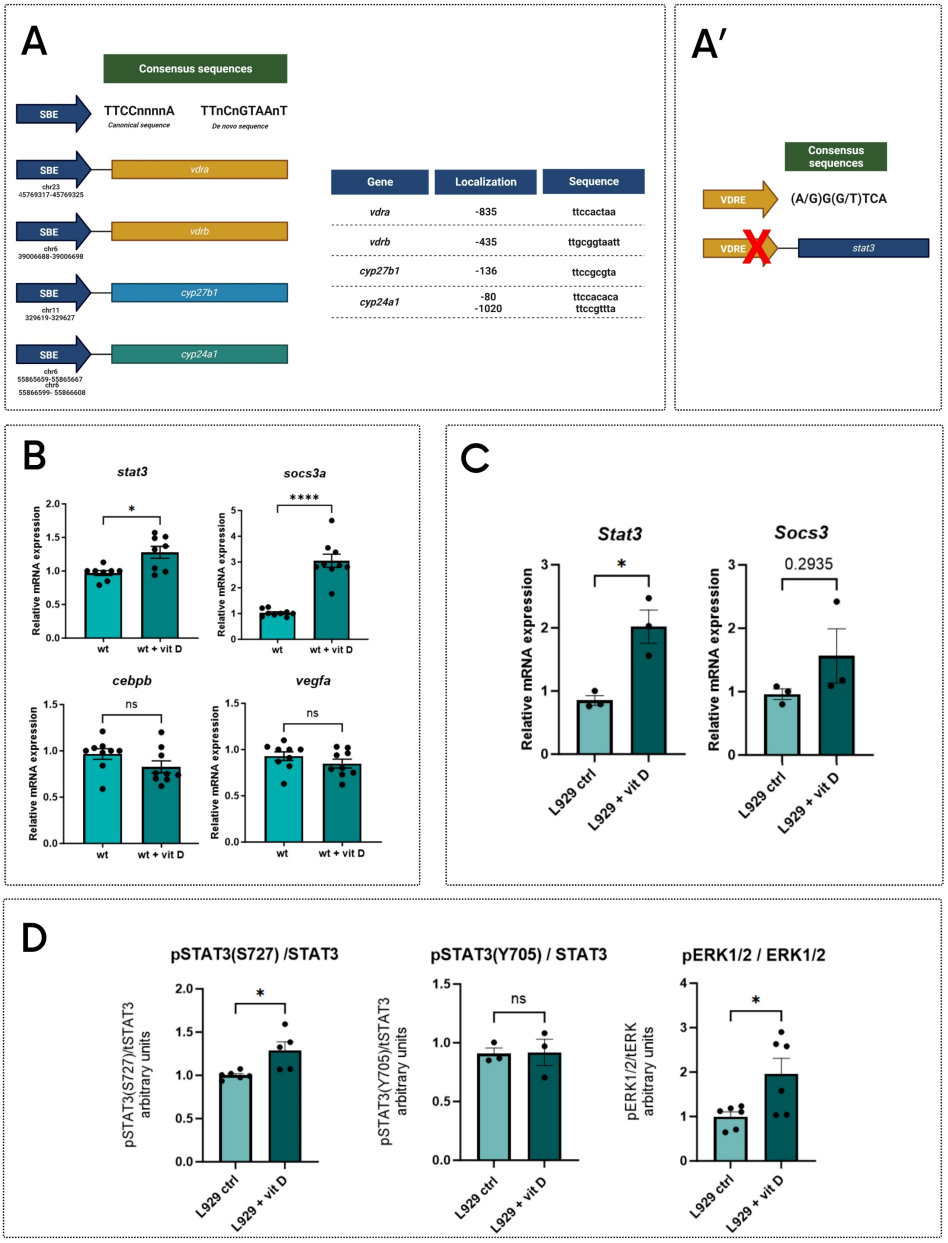
** STAT3-VDR molecular interplay. A** Analysis of sequences of proximal promoter of *vdra*, *vdrb*, *cyp24a1*, *cyp27b1* (A) and *stat3* (A'); representative schemes of SBE (A) and VRE (A') consensus sequences. **B** Expression levels of *stat3*, *socs3a*, *cebpb* and *vegfa* in wild type 5-dpf zebrafish larvae treated for 2 days with vehicle or 0.5 nM vitamin D. **C** Expression level of *Stat3* and *Socs3* in L929 cells treated either with vehicle or 200 nM vitamin D for 24 hours. **D** Protein level quantification of pSTAT3(S727), pSTAT3(Y705) and pERK1/2, (respectively normalized on STAT3, STAT3 and ERK1/2,) of L929 cells treated either with vehicle or 200 nM vitamin D for 24 hours. Mean ± SEM. *p < 0.05, ****p < 0.0001.

As indicated above, *stat3^-/-^* and *stat3^A751/A751^* zebrafish larvae have a reduced expression of genes involved in the vitamin D pathway (**Figure 3A-B**), hence, we analyzed *in silico* the sequences of their proximal promoters. Results showed that *cyp27b1* and c*yp24a1* promoters include canonical Stat3 binding elements (SBE) (**Figure 6A**), indicating that these genes are potential Stat3 targets. In addition, *vdrb* promoter contains a non-canonic SBE (TTnCnGTAAnT) already described in Langlais *et al.* (2012)^43^, whereas the *vdra* promoter includes a SBE element (**Figure 6A**). This evidence suggests that Stat3 may control the expression of *vdra* and *vrdb* by binding their promoter regions.

To verify whether a positive feedback loop between vitamin D and Stat3 is present, we analyzed the expression of *stat3* and *socs3a* genes in 5-dpf whole body samples treated with vitamin D after tail fin amputation. Both genes are upregulated in treated larvae compared to control, suggesting that vitamin D can trigger Stat3 expression and transcriptional activity. At the same time, we could not detect any significant differences in EGFP fluorescence of the *Tg(7xStat3-Hsv.Ul23:EGFP)^ia28^* reporter line (**Figure S5D**) as well as in the expression of Stat3 canonical targets *cebpb* and *vegfa* upon vitamin D treatments, suggesting that vitamin D can regulate Stat3 activity in an uncharacterized way (**Figure 6B**).

The search of vitamin D response elements (VDRE) in the *stat3* proximal promoter region did not give positive results (**Figure 6A'**). This suggests that, while Stat3 can directly modulate the transcription of genes related to the vitamin D pathway, vitamin D probably modulates Stat3 activities by an indirect mechanism as a feedback effect. Trying to better understand the molecular relationship between Stat3 and vitamin D, we studied STAT3 activity in L929 cells upon vitamin D treatment. First, we observed that *Stat3* and *Socs3* expression are upregulated by the treatment (**Figure 6C**), and, whereas STAT3 pY705 levels are not affected, STAT3 pS727 levels are increased by vitamin D treatment (**Figure 6D**). Since ERK pathway is one of the most commonly known agonists of STAT3 pS727 9,44, we decided to measure the levels of pERK1-2/tERK in L929 upon vitamin D treatment. Noteworthy, vitamin D leads to a significant induction of pERK1-2/tERK (**Figure 6D, S6A**), and increases the expression of *mapk1* and* mapk3*, coding for ERK2 and ERK1, respectively (**Figure S6B**). These last pieces of evidence suggest that long-lasting vitamin D treatment might potentiate STAT3 transcriptional activity by inducing MAPK/ERK pathway and, consequently, determining the STAT3 pS727. This phenomenon is in line with the literature proposing that pS727 enhances STAT3 transcriptional activity in specific cellular contexts 4,6,40,45,46.

## Discussion and Conclusion

We demonstrated that the inhibition of STAT3 impairs injury repair in the L929 cell line and we confirmed this result using the tail fin regeneration test in *stat3^-/-^* zebrafish line. As this impairment correlates with a general downregulation of genes involved in the immune response, cell differentiation and vitamin D pathway 47-50, we confirmed that the genes related to these processes are negatively affected by the lack of *stat3*. Unexpectedly, also the newly generated *stat3^A751/A751^* zebrafish line is characterized by both a marked defect in tail fin regeneration and the transcriptional downregulation of the same target genes, suggesting a strong involvement of pS727 in the Stat3-mediated regulation of the regeneration process. Moreover, the lethal effect of the Serine-to-Alanine substitution reveals the importance of pS727 for survival, adding new evidence about the role of this residue in STAT3 functions.

Trying to understand how Stat3 controls regeneration, we identified the genes involved in the metabolism of vitamin D (*cyp27b1* and *cyp24a1*) as interesting targets. Vitamin D exerts many diverse effects in cell homeostasis and renewal, regulating the activity of several signaling pathways. Moreover, vitamin D levels have been correlated with tail fin regeneration of adult zebrafish 22,23. Interestingly, the expression of genes related to vitamin D pathway is significantly downregulated in both *stat3* mutant lines, suggesting an interesting crosstalk between the Stat3 and vitamin D signaling pathways. In this work, we dissected the role of vitamin D in the regeneration of zebrafish larval tail fin, proving that the vitamin D treatment regulates regeneration by promoting the expression of the same regeneration-related genes otherwise downregulated in the *stat3^-/-^* and the *stat3^A751/A751^* mutant lines: indeed, vitamin D partially rescues the regenerative defects observed in *stat3* mutants. On top of that, we demonstrated that vitamin D has a role the regenerating tail fin.

Based on our data, pS727-Stat3 regulates the expression of genes involved in vitamin D metabolism, whereas vitamin D treatment, in turn, increases Stat3 gene transcription, suggesting a complex interplay between the two pathways. Many pieces of evidence about a possible crosstalk between vitamin D and STAT3 pathways have been proposed in literature 51-56, but, the effect of vitamin D levels, or its supplementation, on the STAT3 non-canonical phosphorylation in S727 has never been taken into consideration. We demonstrated that vitamin D administration induces the activation of the MAPK/ERK1-2 pathway, as already observed by Cordes *et al.* 57, which, in turn, triggers the STAT3 pS727 9,10. On the other hand, STAT3 regulates the transcription of vitamin D-related genes in a pS727-dependent fashion, determining a multifaceted interplay between STAT3 and vitamin D pathways.

We hypothesize that pS727 STAT3 regulates the transcription of genes involved in the regeneration processes, including genes coding for the enzymes required for the mature vitamin D synthesis. As a consequence, either the lack of STAT3 or an impairment in S727 phosphorylation, results in a reduction of vitamin D metabolism and, thus, in regeneration. This seems to be correlated not only to the vitamin D mediated activation of VDR transcriptional activity, but also to the VDR ability to increase the pS727 STAT3 levels promoting the MAPK/ERK1-2 pathway as a positive feedback mechanism. This hypothesis is supported by the downregulation of vitamin D-related genes in *stat3* mutants and the upregulation of ERK1-2 upon vitamin D treatment. In other words, bypassing the impairment of vitamin D metabolism through the administration of already active vitamin D, our *stat3* mutants are able to partially recover the VDR-mediated transcription of target genes involved in regeneration. However, the response in the mutants is not comparable to the *wild type* condition probably due to the absence of the feedback loop exerted by vitamin D on MAPK/ERK1-2- dependent stimulation of STAT3 pS727.

Our data are in line with literature that reports the involvement of STAT3 in neovascularization and wound healing after an injury 58-60 and proposes it as a promising target to treat diabetic wounds 61. Accordingly, AD-HIES patients, characterized by loss of function mutations of *STAT3* gene, present a serious impairment in the wound healing process 62. Interestingly, we identified the ability of vitamin D treatment to rescue the regeneration phenotype correlated to *stat3*, proposing vitamin D as a valuable treatment for tissue regeneration impairment of AD-HIES patients.

## Material and Methods

### Maintenance and husbandry of zebrafish lines

Zebrafish were staged and fed as described by Kimmel and colleagues 1995 63 and maintained in large-scale aquaria systems. Embryos were obtained by natural mating, raised at 28°C in Petri dishes containing fish water (50X: 25 g Instant Ocean, 39.25 g CaSO_4_ and 5 g NaHCO_3_ for 1 L) and kept in a 12 h light/12 h dark (LD) cycle. All experimental procedures complied with European Legislation for the Protection of Animals used for Scientific Purposes (Directive 2010/63/EU) and were approved by the Animal Ethics Committee of the University of Padua and by the Italian Ministry of Health (OPBA 23-2015; OPBA 258/2022-PR). Addition of tricaine (MS222; E10521, Sigma-Aldrich) was used for anesthesia or euthanasia of zebrafish embryos and larvae.

In addition to the new mutant lines generated in this work, we used the* stat3^ia23^* mutant line and *Tg(7xStat3-Hsv.Ul23:EGFP)^ia28^* transgenic zebrafish line, described in Peron et *al.*, 2020^14^.

### Generation of *stat3^A751/A751^* and *stat3^A729/A729^* mutant lines and morphological characterization

Following the protocol described in 64, the *stat3^A751/A751^* and *stat3^A729/A729^* were generated by injecting in 1-cell stage embryos the Cas9 protein, the sgRNAs targeting respectively exon 23 and 22 of *stat3* gene and the donor DNA reported in Table S3. Carrier mutants were isolated genotyping the F_1_, for germline transmission of the mutation. Heterozygous mutants harbouring the planned mutation were outcrossed two times with WT and then incrossed to obtain homozygous mutants (F_4_ generation).

Mutants are routinely genotyped using two separate allele-specific PCRs. This analysis requires one common reverse primer to be used with allele-specific forward primers in which the 3' end overlaps with the WT or the mutant sequence. The sequences of these primers are reported in Table S2. The morphological characterization of the larvae was performed following the protocol described in 64.

### Protein extraction and western blotting

Pools of zebrafish larvae and cells were lysed in an appropriate volume of RIPA buffer (20 mM Tris-HCl pH 7.5, 150 mM NaCl, 1 mM EDTA, 0.5 mM sodium pyrophosphate (Na_4_P_2_O_7_), 1 mM β-glycerophosphate (C_3_H_7_Na_2_O_6_P), 1 mM sodium orthovanadate (Na_3_VO_4_)) containing 1% protease inhibitor cocktail (Sigma-Aldrich). Protein concentration was determined with the Pierce BCA Protein Assay Kit following the manufacturer's instructions (Thermo Scientific #23225) and 40 μg of total protein of each sample were prepared for SDS-PAGE with the addition of sample buffer 4×. Electrophoresis was performed using ExpressPlus PAGE precast gels 4-20% (GeneScript), according to the manufacturer's instructions. After electrophoresis, protein samples were transferred on PVDF membranes (Bio-Rad) through a Trans-Blot TurboTM Transfer System (Bio-Rad) in semi-dry conditions, with the 1× transfer buffer (Bio-Rad) at 25 V for 20 min. Proteins were identified by using the appropriate primary antibodies against: STAT3 p705 (Cell Signaling Technology #9145, 1:1000), STAT3 p727 (Cell Signaling Technology #9134, 1:1000), β-actin (Sigma-Aldrich #A1978, 1:10000), Total ERK1/2 (4695, Cell Signaling Technology #4965, 1:1000), pERK 1/2 ((Thr202/Tyr204, Thr185/Tyr187) 12-302, Millipore, 1:1000). PVDF membranes were then incubated for 1 h at RT with appropriate horseradish peroxidase (HRP)-conjugated secondary antibodies (goat a-rabbit IgG-HRP Sigma-Aldrich #A9169, rabbit a-mouse IgG-HRP Sigma-Aldrich #A9044). The signal was revealed using Immobilon Forte Western HRP substrate (Millipore) and the VWR Imager Chemi Premium. Images were acquired in .tiff format and processed by the ImageJ software to quantify the total intensity of each single band.

### Drug treatments

Treatments of zebrafish larvae and cells were performed, at the concentration below reported, with the following compounds: LIF (H17002 Sigma); AG490 (T3434 Sigma); PD98059 (Axon 1386, Axon Medchem); vitamin D (D1530 Sigma); AZD1480 (Selleckchem, Houston, TX); IL-6 (R&D Systems). IL-6 was diluted in PBS and stored at - 80° C. Vitamin D was diluted in ethanol and stored at -20 °C. The other compounds were diluted in DMSO and stored in small aliquots at -20° C.

Larvae were treated: at 3-dpf with AG490 (50 µM) and PD98059 (12.5 µM) for 36h; at 3-dpf with vitamin D (0.5 nM) for 48h; at 3-dpf with LIF (200 nM) for 48h; IL-6 (0.5 ng/ml) for 48 h; AZD1480 (0.1 µM) for 36 h.

Cells were treated: with IL-6 1 ng/ml for 6h; AZD1480 5 μM for 24h; with PD98059 12.5 μM for 24h; vitamin D 0.5 nM for 24h.

### Cell culture

1205Lu melanoma cells were cultured with RPMI, while B16F10 melanoma cells and L929 fibroblast in DMEM. All cell cultures were supplemented with 10% FBS, 100 units/mL penicillin, 0.1 mg/mL streptomycin. Cells were maintained in a humidified 5% CO_2_ atmosphere at 37 °C. The cells were plated at 0.5 × 10^6^ per well in 6-well plates and allowed to adhere overnight. For scratch assays, cells were plated on a 24-well plate at a concentration of 0.3 × 10^6^ per well and allowed to proliferate into a monolayer for 24 h. The monolayer was scratched with a sterile pipet tip (200 μL), washed with serum free RPMI, and photographed with an Olympus IX81 spinning disk microscope (0 h). Cells were then treated with AZD1480 and PD98059. After 24 h, the monolayers were photographed with the same microscope.

### Cytokine quantification

Cytokine concentrations were measured by specific DuoSet ELISAs according to manufacturer's instructions (R&D Systems).

### Imaging

For *in vivo* imaging, larvae were anesthetized with 0.04% tricaine, embedded in 1% low-melting agarose and mounted on a depression slide. Images were acquired using Leica M165 FC microscope equipped with a Nikon DS-Fi2 digital camera. Nikon C2 confocal system was used to acquire images of larvae in *Tg(7xStat3-Hsv.Ul23:EGFP)^ia28^* transgenic background. All images were analyzed with Fiji (ImageJ) software and fluorescence integrated density was calculated setting a standard threshold on non-fluorescent samples.

### mRNA isolation and RT-qPCR

Total RNA was extracted with TRIzol reagent (15596018, Thermo Fisher Scientific) from pools of 10 larvae at 5- or 6-dpf or 20 tails from 5-dpf larvae. RNA samples were treated with RQ1 RNase-Free DNase (M6101, Promega) for elimination of possible genomic DNA contaminations and then used for cDNA synthesis with High-Capacity cDNA Reverse Transcription Kit (4368813, Thermo Fisher Scientific) according to the manufacturer's protocol. qPCRs were performed in triplicate with SybrGreen method on a CFX384 Touch Real-Time PCR Detection System (BioRad) and the 5x HOT FIREPol EvaGreen qPCR Mix Plus (Solis BioDyne, 08-36-00001) following the manufacturer's protocol. *z-actb* and *m-Actb* were used as internal standards in zebrafish and mouse samples, respectively. The amplification protocol consists of 95 °C for 14 min followed by 40 cycles at 95 °C for 15 s, 60 °C for 20 s and 72 °C for 25 s. Threshold cycles (Ct) and melting curves were generated automatically by CFX384 Touch Real-Time PCR Detection System software and results were obtained with the method described in 65. Primer's sequences of the genes of interest were designed with Primer3 software (http://bioinfo.ut.ee/primer3-0.4.0/input.htm) 66 and are listed in Table S1.

### Behavioural assay

The behavioral experiments were performed using the DanioVision^TM^ tracking system (Noldus Information Technology, Wageningen, The Netherlands). Larvae at 6-dpf were placed in 48-well plates with 500 μL of fish water and one larva per well. After 10 min of dark acclimation, movements of larvae were detected and recorded repeating three cycles of 10 min of light and 10 min of dark, as described in MacPhail *et al*. 67.

### Bioinformatic analysis

Promoter analysis was performed on the 2 kb sequence before the start codon of the genes analyzed. Sequences were obtained from Genome Browser Gateway, by University of California Santa Cruz (https://genome.ucsc.edu/cgi-bin/hgGateway) selecting the more recent Zebrafish Assembly (May 2017 (GRCz11/danRer11) (Accession ID: GCA_000002035.4). Consensus sequences were described in **Fig 6A-A'**. The Stat3 alignment was performed using the Clustal Omega software (v1.2.4, https://www.ebi.ac.uk/Tools/msa/clustalo/)^68^.

### Statistical analysis

Statistical analysis was performed using Graph Pad Prism software 10.0.2. Data are presented as the means ± SEM. Comparison between different groups of samples was performed by Student's *t*-test (comparison between two groups) or ordinary/Brown-Forsythe and Welch ANOVA tests (comparison between more than two groups) with a confidence interval of 95%.

## Supplementary Material

Supplementary figures and tables.
