# Supplementary material for: Tail Fin Regeneration in Zebrafish: The Role of Non-canonical Crosstalk Between STAT3 and Vitamin D Pathway
Source: Int J Biol Sci. 2025 Jan 1;21(1):271–84. doi: 10.7150/ijbs.96400 (PMC11667806; doi:10.7150/ijbs.96400)
Supplement: Supplementary file 1 — Supplementary figures and tables. [file ijbsv21p0271s1.pdf]

Supplementary materials

Supplementary images

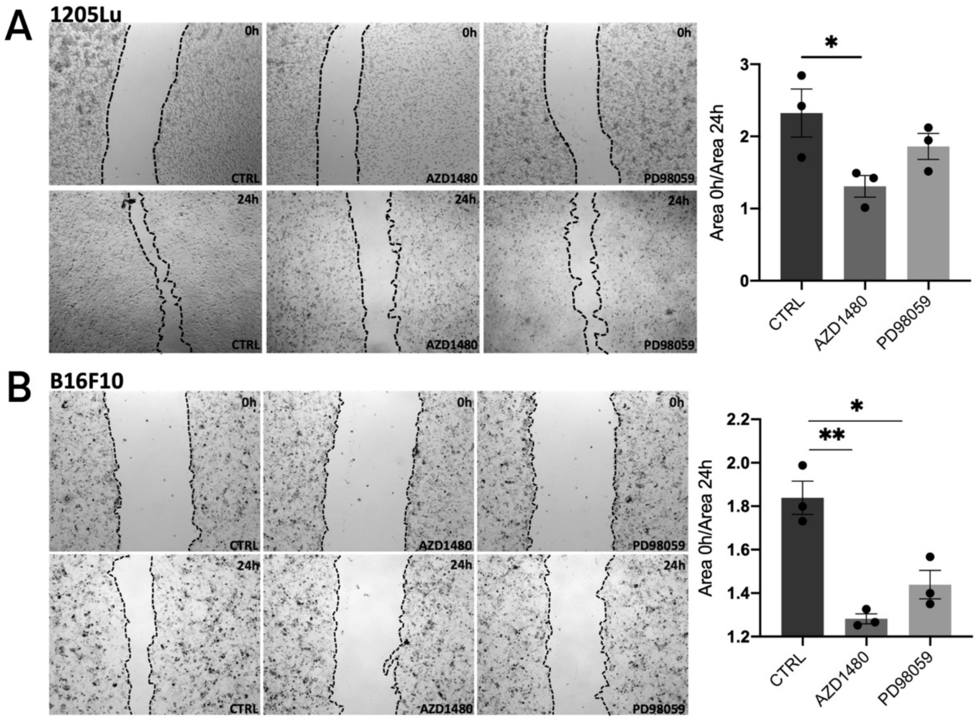

Figure S1. STAT3 inhibition hampers scratch reoccupation in melanoma cell cultures. A Representative pictures and relative quantification of 1205Lu cells treated with vehicle, 5 µM AZD1480 or 12.5 µM PD98059 for 24 hours. **B** Representative pictures and relative quantification of B16F10 cells treated with vehicle, 5 µM AZD1480 or 12.5 µM PD98059 for 24 hours. Mean ± SEM. * $p < 0.05$, **$p < 0.01$.

```
Danio       GNGGRTNSDASLIVTEELHLITFETEVYHQGLKIDLETHSLPVVVISNICQMPNAWASIL    479
Xenopus     GNGGRANCDASLIVTEELHLITFETEVYHQGLKIDLETHSLPVVVISNICQMPNAWASIL    478
Homo        GNGGRANCDASLIVTEELHLITFETEVYHQGLKIDLETHSLPVVVISNICQMPNAWASIL    478
Mus         GNGGRANCDASLIVTEELHLITFETEVYHQGLKIDLETHSLPVVVISNICQMPNAWASIL    478
            *****:*.****************************************************

Danio       WYNMLTNHPKNVNFFTKPPVGTWDQVAEVLSWQFSSTTKRGLTIEQLTTLAEKLLGPCVN    539
Xenopus     WYNMLTNNPKNVNFFTKPPIGTWDQVAEVLSWQFSSTTKRGLSIEQLTTLAEKLLGPGVN    538
Homo        WYNMLTNNPKNVNFFTKPPIGTWDQVAEVLSWQFSSTTKRGLSIEQLTTLAEKLLGPGVN    538
Mus         WYNMLTNNPKNVNFFTKPPIGTWDQVAEVLSWQFSSTTKRGLSIEQLTTLAEKLLGPGVN    538
            *******:***********:**********************:************** **

Danio       YSGCQITWAKFCKENMAGKGFSFWVWLDNIIDLVKKYILALWNEGYIMGFISKERERAIL    599
Xenopus     YSGCQITWAKFCKENMAGKGFSFWVWLDNIIDLVKKYILALWNEGYIMGFISKERERAIL    598
Homo        YSGCQITWAKFCKENMAGKGFSFWVWLDNIIDLVKKYILALWNEGYIMGFISKERERAIL    598
Mus         YSGCQITWAKFCKENMAGKGFSFWVWLDNIIDLVKKYILALWNEGYIMGFISKERERAIL    598
            ************************************************************

Danio       SPKPPGTFLLRFSESSKEGGITFTWVEKDINGKTQIQSVEPYTKQQLNSMSFAEIIMGYK    659
Xenopus     STKPPGTFLLRFSESSKEGGITFTWVEKDISGKTQIQSVEPYTKQQLNNMSFAEIIMGYK    658
Homo        STKPPGTFLLRFSESSKEGGVTFTWVEKDISGKTQIQSVEPYTKQQLNNMSFAEIIMGYK    658
Mus         STKPPGTFLLRFSESSKEGGVTFTWVEKDISGKTQIQSVEPYTKQQLNNMSFAEIIMGYK    658
            * ******************:*********.*****************.***********

Danio       IMDATNILVSPLVYLYPDIPKEEAFGKYCRPETHPDTEFPDTGCVTQPYLKTKFICVTPC    719
Xenopus     IMDATNILVSPLVYLYPDIPKEEAFGKYCRPESQEHQEPTDPG-STAPYLKTKFICVTPT    717
Homo        IMDATNILVSPLVYLYPDIPKEEAFGKYCRPESQEHPE-ADPG-SAAPYLKTKFICVTPT    716
Mus         IMDATNILVSPLVYLYPDIPKEEAFGKYCRPESQEHPE-ADPG-SAAPYLKTKFICVTPT    716
            ********************************::  .*  * *  : ************

Danio       PSVFMDFPDSELLGNGFPGTNSGNTSDLFPMSPRTLDSLMHNEA-----AEANPGPLESL    774
Xenopus     TCS---------------------STLDLPMSPRTLDSLMQFP--GEGADSSAGNQFETL    754
Homo        TCS---------------------NTIDLPMSPRTLDSLMQFGNNGEGAEPSAGGQFESL    755
Mus         TCS---------------------NTIDLPMSPRTLDSLMQFGNNGEGAEPSAGGQFESL    755
             .                      .:  :***********:          :  . :*:*

Danio       TLDMELSSDHASPMREGFAASTVSDMDTCRNA   806
Xenopus     TFDMELTSECASSPM-----------------   769
Homo        TFDMELTSECATSPM-----------------   770
Mus         TFDMDLTSECATSPM-----------------   770
            *:**:*:*: *:
```

Figure S2. Alignment of *Danio rerio*, *Xenopus laevis*, *Mus musculus* and *Homo sapiens* STAT3 amino acid sequences. The Y708, S729 and S751 phosphorylation sites are respectively highlighted in blue, orange and green. S729 belongs to a zebrafish-specific domain, whereas zebrafish S751 corresponds to S727 in frog, mouse and human.

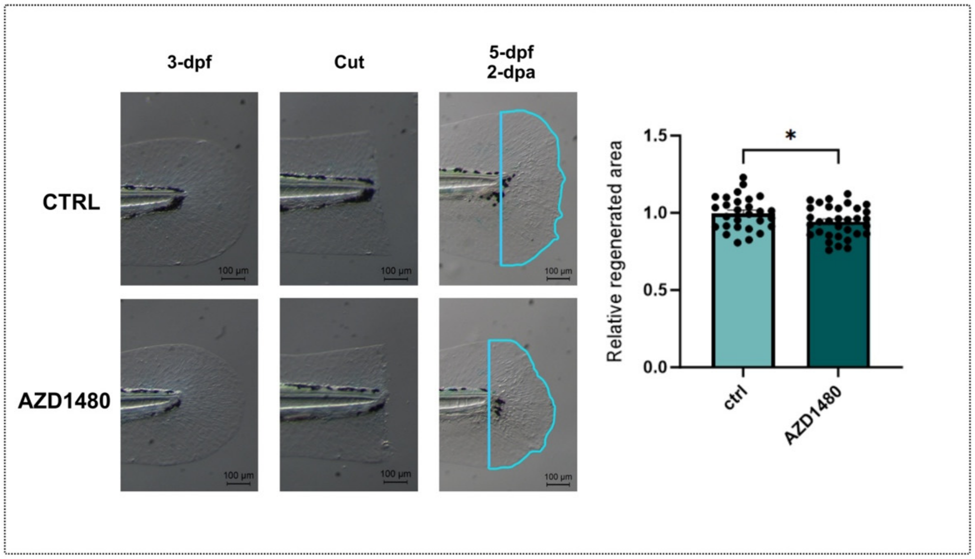

Figure S3. Representative pictures and quantification of regenerated area of wild type zebrafish tail fins cut at 3-dpf and treated for 2 days either with vehicle or 0.1 µM AZD1480. Mean ± SEM. * $p < 0.05$.

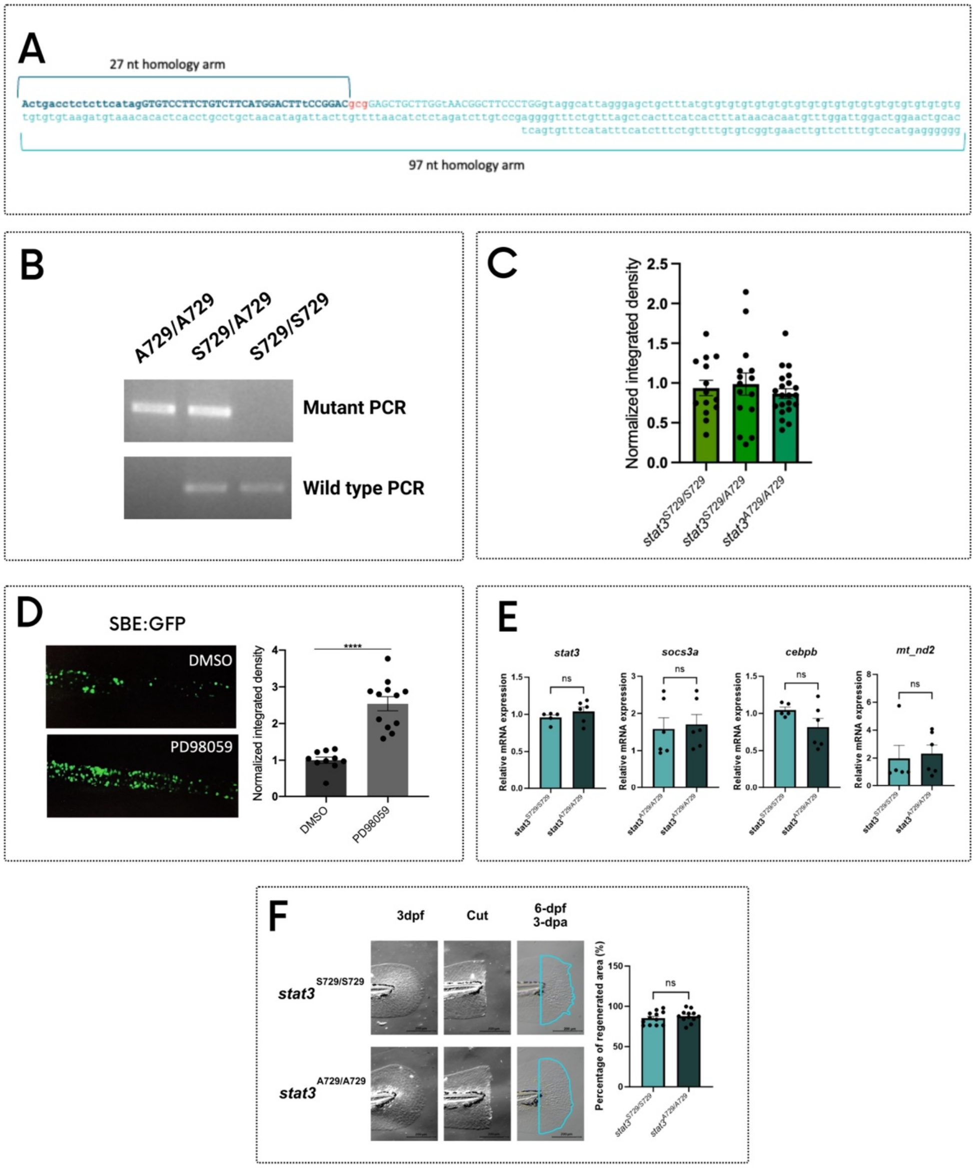

Figure S4. Generation of *stat3*$^{S\rightarrow A729}$ zebrafish knock-in line. A Sequence of the donor DNA used to generate the KI. The mutation (gcg) is highlighted in red and is flanked by a 27-nt and a 97-nt homology arms. **B** Representative picture of allele-specific PCRs routinely performed to genotype KI fish: heterozygotes are positive for both alleles, whereas homozygotes are positive for only one. **C** Fluorescence quantification of *Tg(7xStat3-Hsv.Ul23:EGFP)*^{ia28} intestines of 6-dpf *stat3*$^{S729/S729}$, *stat3*$^{S729/A729}$ and *stat3*$^{A729/A729}$ larvae. Scale bar: 100 µm. **D** Fluorescent image of *Tg(7xStat3-Hsv.Ul23:EGFP)*^{ia28} intestines of 6-dpf wild type larvae treated either with DMSO or 12.5 µM PD98059 for 3 days. Scale bar: 100 µm. **E** Expression level of *stat3*, *socs3a*, *cebpb*, and *mt-nd2* in 6-dpf *stat3*$^{S729/S729}$ and *stat3*$^{A729/A729}$ larvae. **F** Regeneration rate of 6-dpf *stat3*$^{S729/S729}$ and *stat3*$^{A729/A729}$ larvae at 3 dpa. Mean ± SEM. ns = not significant.

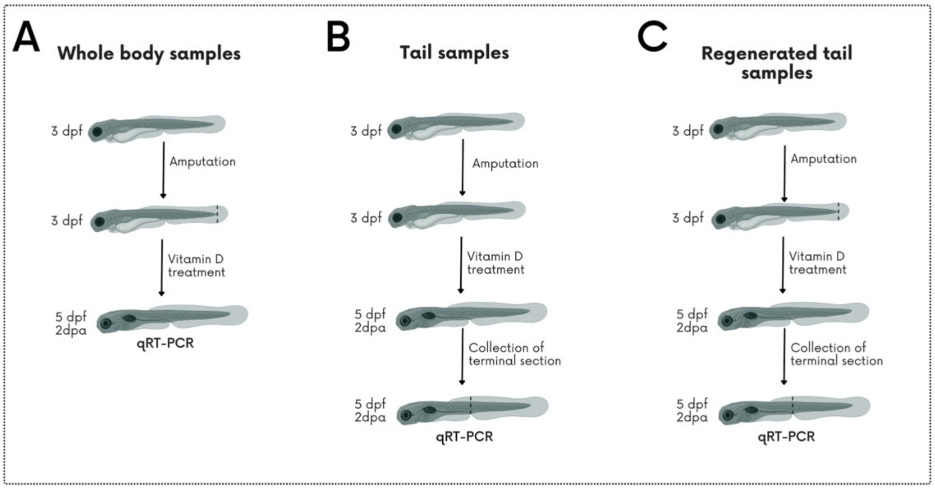

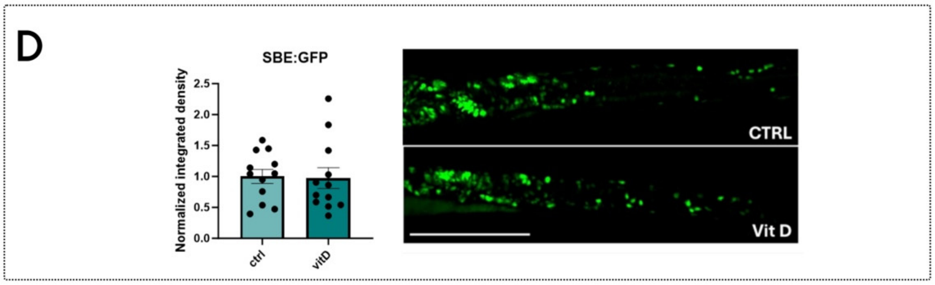

Figure S5. Schematic representation of samples collected for vitamin D effects on zebrafish larvae. **A** Whole body samples: we cut 3-dpf zebrafish larvae tail fins and we treated them for 2 days with 0.5 nM vitamin D, at the end of the treatment larvae were homogenized to extract RNA. **B** Tail samples: we treated 3-dpf larvae with 0.5 nM vitamin D for 2 days and at the end of the treatment we cut the tail (from the anus to the distal part of tail fin) of each treated larva; we pooled 20 tails for each condition. **C** Regenerated tail samples: we cut 3-dpf zebrafish larvae tail fins and we treated them for 2 days with 0.5 nM vitamin D, at the end of the treatment we cut the tail (from the anus to the distal part of tail fin) of each treated larva; we pooled 20 tails for each condition. **D**. Fluorescence quantification of *Tg(7xStat3-Hsv.Ul23:EGFP)^{ia28}* intestines of 5-dpf treated with vitamin D for 48h and relative control. Scale bar: 200 µm. Mean ± SEM.

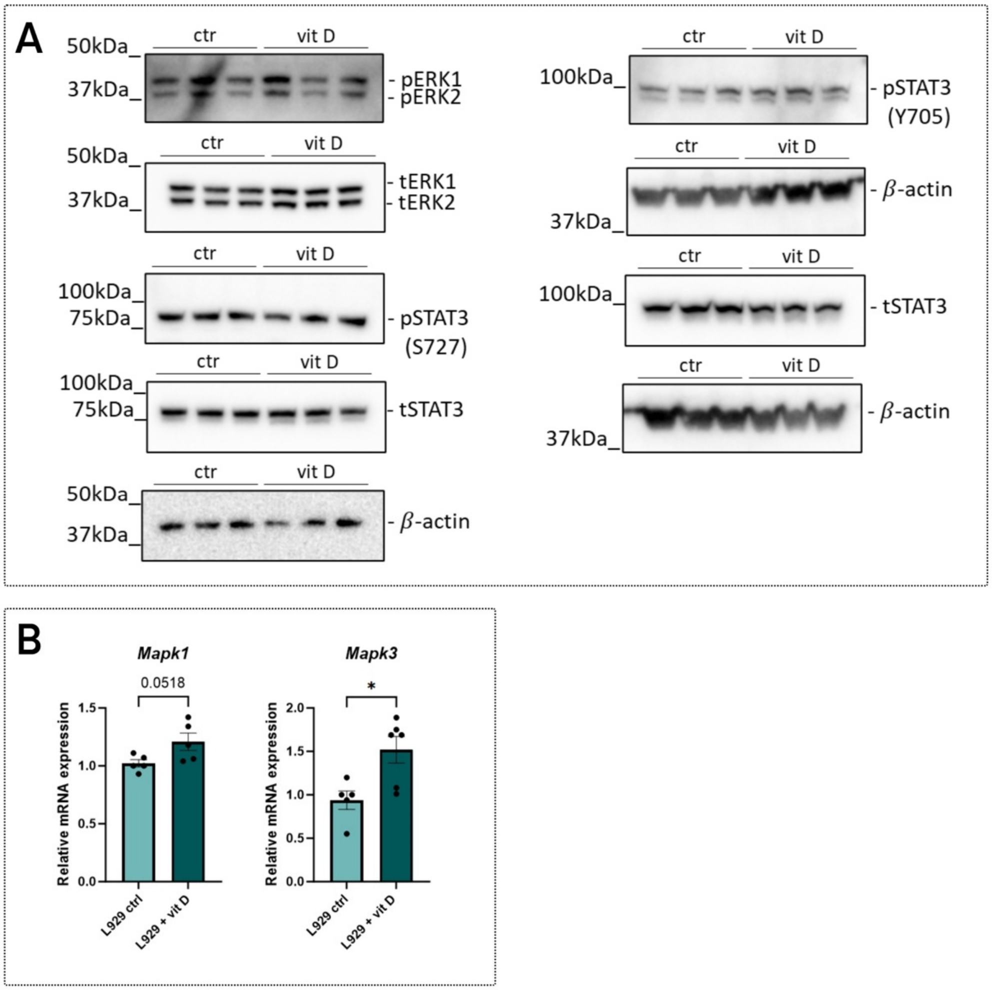

Figure S6. Western blot images. A Western blot analysis of ERK 1/2, pERK1/2, STAT3, pSTAT3 (Y705 and S727) protein in L929 cells treated either with vehicle or 200 nM vitamin D for 24 hours. n=3 biological replicates. **B** Expression level of *Mapk1* and *Mapk3* in L929 cells treated either with vehicle or 200 nM vitamin D for 24 hours. Mean ± SEM. * $p < 0.05$.

Supplementary tables

Table 1. Primers for RT-qPCR

Gene	Forward	Reverse
z-actb	TGGGTATGGAATCTTGCGGT	GTGGGGCAATGATCTTGATCT
z-stat3	TGCCACCAACATCCTAGTGT	GCTTGTTTGCACTTTTGACTGA
z-socs3a	GGAAGACAAGAGCCGAGACT	GCGATACACACCAAACCCTG
z-vegfa	AAAAGAGTGCGTGCAAGACC	TTTCGTGTCTCTGTCGGGAC
z-cebpb	CCAAAAGTAACGGGCGACAC	ATCTTCCCTTACCTGACGGC
z-ucmaa	GTTTTCGTGCCAGCATCTG	GTGTTCGTTGCTCTGTTCCT
z-cyp26b1	GCTGTCAACCAGAACATTCCC	GGTTCTGATTGGAGTCGAGGC
z-sp7	AACCCAAGCCCGTCCCGACA	CCGTACACCTTCCCGCAGCC
z-mpx	AGGTGTTGCTGAGCCTTTTG	ACCACAACCTATCGCCATCT
z-il4	GCAGGAATGGCTTTGAAGGG	TCCTTCATTGTGCATTCCCC
z-il21	AGTGCAAATCATGTGAAGCGT	GACTCTTCAGGTCTCTACGCT
z-cyp24a1	GCCTGTTGAGCTCCACAAAA	CTGCAGGTTCTTGTCGATGT
z-vdrb	AGACTCAAGCGTTGCCTAGA	GCCTCCTCATCCTTCCTTCT
z-vdra	GGACCAGACTTCAAATACTGCA	TCAGCTTCTTCAGACCAACCT
z-rarga	ATTCCGCCAGAGAGCTATGA	TAGGCCCAGGTCTAGCTGAA
z-mt-nd2	GCAGTAGAAGCCACCACAAA	GCTAGACCGATTTTGAGAGCC

m-Actb	CTAAGGCCAACCGTGAAAAG	ACCAGAGGGCATACAGGGACA
m-Stat3	TGTTGGAGCAGCATCTTCAG	GAGGTTCTCCACCACCTTCA
m-Socs3a	ATTTCGCTTCGGGACTAGC	AACTTGCTGTGGGTGACCAT
m-Mapk1	CGCTACACCAACCTCTCGTA	AGGTCTGGTGCTCAAAAGGA
m-Mapk3	ATAGGCATCCGAGACATCCT	ATGTGGTCATTGCTCAGCTG

Table 2. Primers for genotyping PCR

Locus	Forward	Reverse
stat3 ex14	GGCCTCTCTGATAGTGACCG	AGTTGTGCTTAGACGCGATC
stat3 ex23	GCGATTTGTTCCCAATGTCG (FW WT)<br>GCGATTTGTTCCCAATGgca (FW AA)	CGTCATGACTCACCGAGAGG
stat3 ex22	TCATGGACTTCCCGGACAGT (FW WT)<br>TCATGGACTTtCCGGACgcg (FW AA)	CCCCCTCATGGACAAAAGAAC

Table 3. Sequences of gRNA used to generate *stat3* KI lines

Locus	Forward
stat3 ex23	AUGAGAGAGUCGAGCGUGCG
stat3 ex22	AGUGAGCUGCUUGGGAA
